# Deep Abscess of the Post-Cervical Region, with Sudden Death

**Published:** 1884-02

**Authors:** B. G. Long


### Deep Abscess of the Post-Cervical Region, with Sudden Death.

Reported by B. G. Long, M. D.

Anna Z——, aged 12, of sound health and good family history, attended school regularly until the afternoon of December 17, 1883, when, on account of stiffness and soreness of the back of her neck, she remained at home. The family physician, Dr. Harrington, who was at the house to see another member of the family, suffering with bronchitis, had his attention called to the condition of her neck. But little was thought of it, as she was working about the house and only slightly inconvenienced.

Three days later, December 20th, he was called to see her, and found her neck, and especially the back of it, enormously swollen. It was very tender to touch, and she was suffering severe pain. Pulse 112; temperature 101°.

Poultice applied and anodyne administered. She grew rapidly worse. The swelling became more circumscribed and harder on the back of the neck. Consultation was held on the 22d, when a hypodermic needle was introduced into the tumor, but no pus found. Brain symptoms were very marked at this time, and an unfavorable prognosis was given. The only symptom of paralysis was a deviation of the tongue to the right side when protruded. Death occurred December 24th, at 1.30 P. M.

The consent of the parents to an autopsy was gained only by promising to open nothing but the back of the neck.

Autopsy twenty-two hours after death. Body moderately well nourished; rigor mortis marked; very great hypostatic congestion of back; a denuded surface on nape of neck where a blister had been applied. Nothing but marked congestion was found down to the spinous processes. Bulging from between the laminæ of the second and third vertebræ, mostly on the right side, was a fluctuating tumor which contained about one and a half drachms of pus. On removing the arch of the second vertebra, the dura mater was found intact; opening into the sub-arachnoid space disclosed a very large amount of serous effusion, the result of meningitis, which was the immediate cause of death.

